# Functional and structural lesion network mapping in neurological and psychiatric disorders: a systematic review

**DOI:** 10.3389/fneur.2023.1100067

**Published:** 2023-06-30

**Authors:** Fardin Nabizadeh, Mohammad Hadi Aarabi

**Affiliations:** ^1^Neuroscience Research Group (NRG), Universal Scientific Education and Research Network (USERN), Tehran, Iran; ^2^School of Medicine, Iran University of Medical Sciences, Tehran, Iran; ^3^Department of Neuroscience and Padova Neuroscience Center (PNC), University of Padova, Padua, Italy

**Keywords:** lesion network mapping, connectivity, lesions, stroke, network, localization

## Abstract

**Background:**

The traditional approach to studying the neurobiological mechanisms of brain disorders and localizing brain function involves identifying brain abnormalities and comparing them to matched controls. This method has been instrumental in clinical neurology, providing insight into the functional roles of different brain regions. However, it becomes challenging when lesions in diverse regions produce similar symptoms. To address this, researchers have begun mapping brain lesions to functional or structural networks, a process known as lesion network mapping (LNM). This approach seeks to identify common brain circuits associated with lesions in various areas. In this review, we focus on recent studies that have utilized LNM to map neurological and psychiatric symptoms, shedding light on how this method enhances our understanding of brain network functions.

**Methods:**

We conducted a systematic search of four databases: PubMed, Scopus, and Web of Science, using the term “Lesion network mapping.” Our focus was on observational studies that applied lesion network mapping in the context of neurological and psychiatric disorders.

**Results:**

Following our screening process, we included 52 studies, comprising a total of 6,814 subjects, in our systematic review. These studies, which utilized functional connectivity, revealed several regions and network overlaps across various movement and psychiatric disorders. For instance, the cerebellum was found to be part of a common network for conditions such as essential tremor relief, parkinsonism, Holmes tremor, freezing of gait, cervical dystonia, infantile spasms, and tics. Additionally, the thalamus was identified as part of a common network for essential tremor relief, Holmes tremor, and executive function deficits. The dorsal attention network was significantly associated with fall risk in elderly individuals and parkinsonism.

**Conclusion:**

LNM has proven to be a powerful tool in localizing a broad range of neuropsychiatric, behavioral, and movement disorders. It holds promise in identifying new treatment targets through symptom mapping. Nonetheless, the validity of these approaches should be confirmed by more comprehensive prospective studies.

## Introduction

For many years, the method of understanding the function of a specific brain region was through the study of focal brain lesions that occurred as a result of strokes, tumors, and hemorrhages. If patients with similar symptoms had overlapping lesions in a specific brain region, we could pinpoint those neurological symptoms or behavioral deficits to that region (1, 2). Traditional lesion mapping has been the cornerstone of clinical neurology, providing valuable insights into the functional roles of different brain regions. However, most neurological and psychiatric symptoms cannot be traced back to a single region; instead, they involve a network of interconnected regions (3). Additionally, patients exhibiting similar symptoms may have lesions located in diverse areas, which poses a challenge to traditional lesion mapping in pinpointing those symptoms (4). Such symptom overlap could potentially be due to the disruption of an underlying, cohesive brain network (5, 6).

One strategy to address this challenge is through the implementation of lesion network mapping (LNM), a methodology that connects brain lesions to either functional or structural networks to identify common brain circuits tied to diverse lesion locations (4, 7).

**Figure 1:**
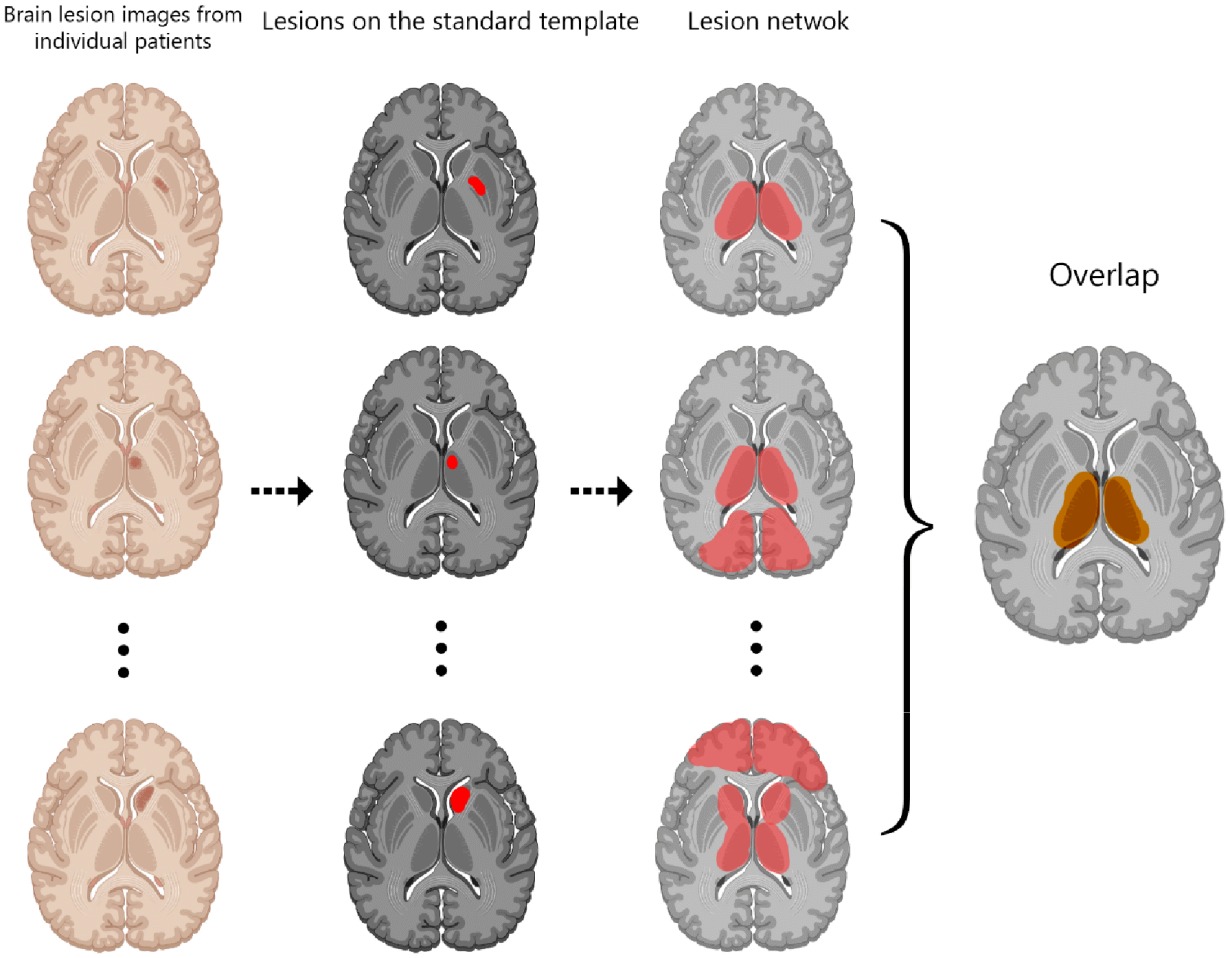
Lesion network mapping procedure.

This technique operates on the hypothesis that a lesion present at any location within a network mapped for a specific symptom has the potential to trigger that symptom (4, 8). The process of performing LNM overlap studies comprises four stages: Firstly, lesions are traced into a standardized brain atlas, establishing a foundation for connectivity analyses (9) (Figure 1). Secondly, an assessment is undertaken to determine the connectivity of each lesion location with other brain regions, using either structural or functional normative human connectome data. Thirdly, correlations between lesion locations and all other brain voxels are thresholded to delineate a map of interconnected regions. Finally, these maps from each patient are superimposed to pinpoint brain regions that are most frequently connected to lesion locations associated with the symptom in question (10). In addition, certain studies incorporate voxelwise statistical analyses, either by leveraging continuous outcomes or by contrasting patients with controls, with the aim of elucidating common networks associated with symptomatic lesions (11, 12). The availability of large-scale functional and anatomical normative maps, like those offered by the Human Connectome Project data, provide a robust foundation for correlating lesion locations with a shared network, thereby facilitating the study of neurological and psychiatric symptoms (13).

This methodology has been broadly utilized since its introduction in 2015 for the localization of an array of neuropsychiatric, behavioral, and movement disorders (8, 10, 14–18). Previous investigations have substantiated the validity of LNM; while several outcomes confirmed primary hypotheses concerning the neuroanatomical underpinnings of specific symptoms (19), some unexpected findings also emerged from LNM studies. For instance, a notable association between the putamen and hemichorea-hemiballism was identified, despite earlier evidence suggesting the involvement of the subthalamic nucleus in the genesis of hemichorea-hemiballism (20). As such, LNM may offer an innovative avenue to further investigate the functionality of brain networks.

The intent of this systematic review is to spotlight recent studies that have utilized LNM to map neurological and psychiatric symptoms, thereby providing insight into how this methodology enhances our comprehension of the function of distinct brain regions.

## Methods and materials

This systematic review was conducted in adherence to the Preferred Reporting Items for Systematic Reviews and Meta-Analyses (PRISMA) guidelines (21).

### Literature search and eligibility criteria

A literature search was performed on PubMed, Scopus, Embase, and Web of Science in June 2022 using the term “Lesion network mapping.” In addition, we manually reviewed the reference lists of pertinent review studies to identify relevant research. Inclusion criteria encompassed observational studies on LNM in neurological and psychiatric disorders. Exclusions were made for case reports, review articles, and non-English language studies.

### Study selection

Two researchers (F.N, M.A) independently examined the titles and abstracts and eliminated irrelevant studies. Subsequently, the remaining articles’ full texts were scrutinized, and studies were selected based on our eligibility criteria.

### Data extraction

Data was collated from the selected studies using a pre-designed data sheet. The collected information included: author, publication year, study design, data source, sample size, age, gender distribution, study duration, underlying disease, lesion cause, lesion location, lesion type, investigated symptom or disorders, number of subjects with investigated symptoms or disorders, analytical software used, normative data, and LNM findings.

### Quality assessments

The quality of the included studies was evaluated using the Newcastle-Ottawa scale (NOS) for observational studies, which operates on a scoring range from 0 to 8 (22).

## Results

### Search results

**Figure 2:**
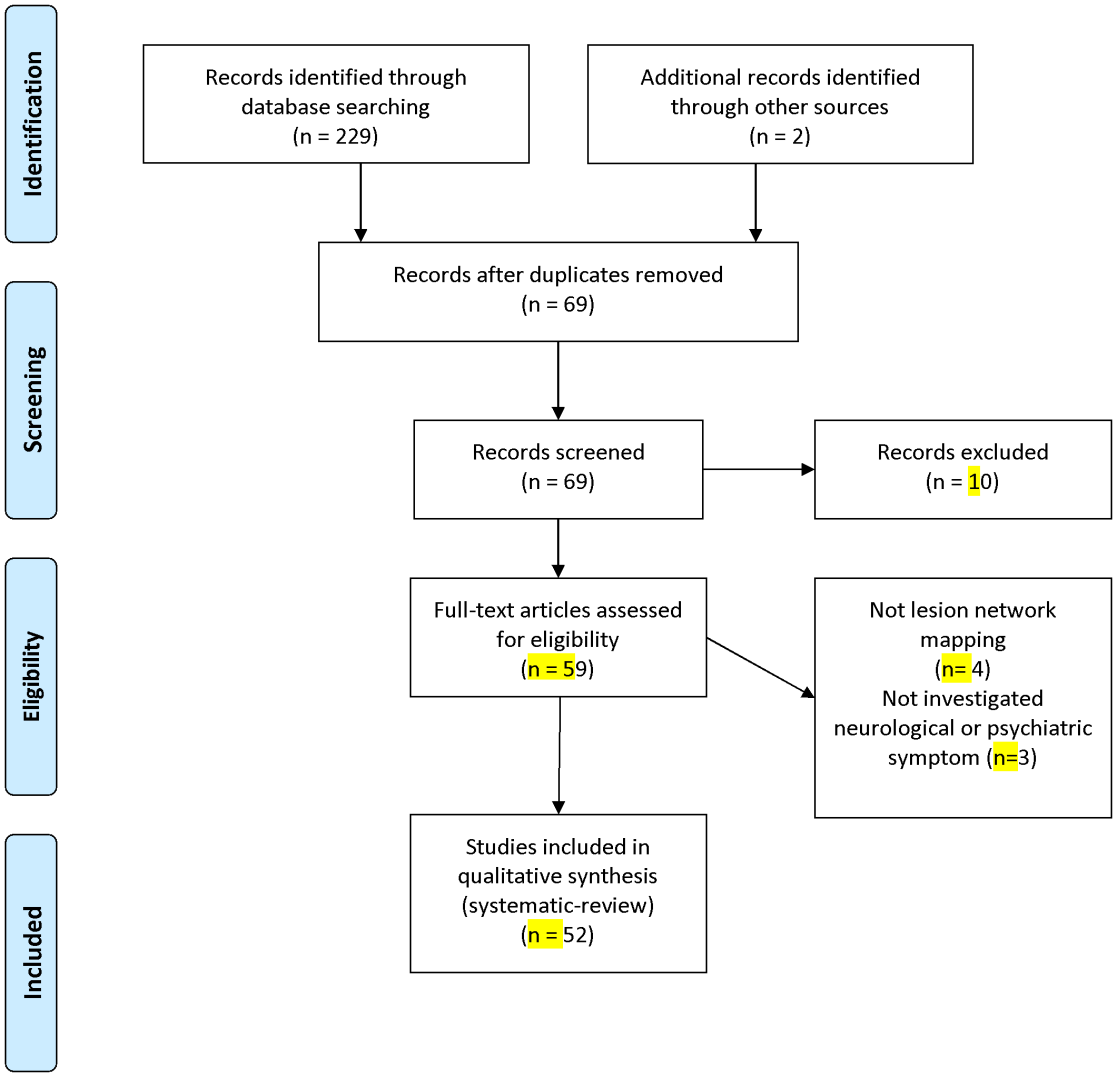
PRISMA flow diagram depicting the flow of information through the different phases of a systematic review.

Our literature search and additional records resulted in 69 studies after eliminating duplicates (Figure 2). Post title and abstract screening, 10 studies were excluded. Ultimately, a total of 52 studies involving 6,814 subjects were included in our systematic review following a full-text review (4–8, 10–12, 14–20, 23–57).

Among the incorporated studies, 39 were cross-sectional, ten were case–control, two were cohort studies, and the remaining one was a longitudinal study. In terms of data sources, 30 studies utilized private data, 20 used published case reports, and two studies employed both private data and published case reports. Notably, 41 studies utilized FSL as their analytical software. A detailed overview of the characteristics of the included studies can be found in Table 1. Among the analyzed studies, functional LNM was performed in 38, structural LNM was conducted in 7, and both forms of LNM were employed in 7 studies. The quality assessment indicated that the mean NOS score of the included studies was 7.48.

**Table 1:** Charcteristics and imaging findings of the included studies.

Author	Year	Study design	Source of data	Sample size	Age	Females	Period of study	Underlying disease	Cause of lesion	Location of the lesions	Type of lesion	Studied symptom or disorder	Number of subjects with symptom	Software	Lesion network mapping	Normative data	NOS
Souter	2022	Cross-sectional	Private	23	62.2 ± 11.9	NR	NR	Stroke	Stroke	Heterogenous	Infarction	Semantic Aphasia	23	MATLAB	Functional and structural	Brain Genomics Superstruct Project (GSP) with 191 healthy participants	7
Bowren	2022	Cross-sectional	Private	593	53–61	324	NR	Stroke	Stroke	NR	Ischemic	Cognitive and motor outcomes	593	FSL	Functional and structural	Human Connectome Project (HCP) with 303 healthy participants	8
Crockett	2022	Cross-sectional	Private	160	74.62	99	NR	Cerebral small vessel disease	Cerebral small vessel disease		Ischemic	Fall risk	160	FSL	Functional	Brain Genomics Superstruct Project (GSP) with 1000 healthy participants	7
Ganos	2022	Cross-sectional	Case reports	22	25.3 ± 20.7	NR	Until 2020	Heterogenous	Heterogenous	Basal ganglia, temporal and parietal lobes, the insula, corpus callosum, thalamus, internal capsule, midbrain, pons and medulla oblongata.	Heterogenous	Tics	22	FSL	Functional	Brain Genomics Superstruct Project (GSP) with 1000 healthy participants	8
Joutsa	2022	Case-control	Private	129	33.7	58	NR	Brain injury	NR	Heterogenous	NR	Addiction	129	FSL	Functional and structural	Brain Genomics Superstruct Project (GSP) with 1000 healthy participants	8
Blondiaux	2021	Cross-sectional	Private	26	NR	NR	NR	Focal epilepsy	Focal epilepsy	NR	NR	Autoscopic phenomena	26	FSL	Functional	Brain Genomics Superstruct Project (GSP) with 98 healthy participants	6
Cohen	2021	Cohort	Private	123	2.66	60	NR	Tuberous sclerosis	Tuberous sclerosis		Tumoral	Infantile Spasms	74	FSL	Functional	Brain Genomics Superstruct Project (GSP) with 1000 healthy participants	8
Crockett	2021	Cross-sectional	Private	160	74.62	99	NR	Cerebral small vessel disease	Cerebral small vessel disease		Ischemic	Global cognition	160	FSL	Functional	Brain Genomics Superstruct Project (GSP) with 1000 healthy participants	7
Germann	2021	Cohort	Private	11	23–44	6	NR	Obsessive compulsive disorder patients underwent focused ultrasound capsulotomy	NR	NR	NR	Obsessive compulsive disorder	11	MATLAB	Functional	Brain Genomics Superstruct Project (GSP) with 1000 healthy participants	7
Higashiyama	2021	Cross-sectional	Case reports	25	37–72	16	Until 2017	Ischemic stroke and brain tumor	Infarction and tumor		Infarct, tumoral	Foreign accent syndrome	25	FSL	Functional	Brain Genomics Superstruct Project (GSP) with 1000 healthy participants	8
Pini	2021	Cross-sectional	Private	123	53	63	NR	Stroke	Stroke	NR	NR	Behavioral deficits	123	FSL	Functional	Brain Genomics Superstruct Project (GSP) with 176 healthy participants	7
Cotovio	2020	Cross-sectional	Case reports	505	54.9 ± 17.7	NR	Until 2017	Heterogenous	NR	Wide range of cortical and subcortical areas	Infarct	Mania	15	FSL	Functional	Brain Genomics Superstruct Project (GSP) with 1000 healthy participants	8
Hwang	2020	Case-control	Private	49	54.8	24	NR	Patients with neurological disorders	Ischemic or hemorrhagic stroke	Thalamus	Ischemic and infarct	Executive Function	15	FSL	Functional	Brain Genomics Superstruct Project (GSP) with 303 healthy participants	8
Klingbeil	2020	Case-control	Private	49	NR	NR	NR	Stroke	Stroke	Heterogenous	Ischemic	Anosognosia for hemiplegia	25	SPM	Functional	NR	6
Kyeong	2020	Cross-sectional	Private	39	67.3 ± 2.6	NR	NR	Stroke	Stroke	Heterogenous	Ischemic	Asymmetric step length	39	FSL	Functional and structural	Brain Genomics Superstruct Project (GSP) with 1000 healthy participants and Cambridge Centre for Ageing and Neuroscience (Cam-CAN)	7
Mansouri	2020	Case-control	Private and case reports	51	NR	23	NR	Tumor	Tumor	Frontal, temporal, and parietal	Tumoral	Epilepsy	51	MATLAB	Functional	Brain Genomics Superstruct Project (GSP) with 1000 healthy participants	7
Philippi	2020	Case-control	Private	48	60	25	NR	Brain injury	Brain injury	Heterogenous	NR	Mind-wandering	29	FSL	Functional	Brain Genomics Superstruct Project (GSP) with 98 healthy participants	8
Salvalaggio	2020	Cross-sectional	Private	132	49.8 ± 9.0 years (visual left) and 54.9 ± 11.9 years (motor right limbs)	NR	NR	Stroke	Stroke	NR	NR	Behavioral deficits	132	FSL	Functional and structural	Human Connectome Project (HCP) with 176 healthy participants	8
Snider	2020	Case-control	Private	171	58	NR	NR	NR	NR	NR	NR	Loss of consciousness	171	FSL	Functional	Brain Genomics Superstruct Project (GSP) with 1000 healthy participants	8
Albazron	2019	Case-control	Private	195	6.8 ± 4.2	84	1988–2017	Pediatric patients who underwent cerebellar tumor resection	Tumor	Medulloblastoma, ependymoma, and astrocytoma/glioma	Tumoral	Cerebellar cognitive affective syndrome	48	FSL	Functional	Brain Genomics Superstruct Project (GSP) with 98 healthy participants	7
Cohen	2019	Cross-sectional	Case reports	44	39	12	2000–2019	Stroke	Stroke	Right fusiform face area		Prosopagnosia	44	FSL	Functional	Brain Genomics Superstruct Project (GSP) with 1000 healthy participants	8
Corp	2019	Cross-sectional	Case reports	25	23–84	11	Until 2017	Heterogenous	Heterogenous	Cerebellum, pons, midbrain, thalamus, globus pallidus interna, basal ganglia, putamen	Haemorrhage (*n*=7), Infarct (*n*=10), Cyst (*n*=3), Tumour (*n*=2), MS plaques (*n*=1), Glioma (*n*=1), and Angioma (*n*=1)	Idiopathic cervical dystonia	25	FSL	Functional	Brain Genomics Superstruct Project (GSP) with 1000 healthy participants	8
Joutsa	2019	Cross-sectional	Case reports	36	NR	NR	Until 2016	NR	Ischemi or hemorrhagi	Midbrain, cerebellum, basal ganglia, pons, medulla, cere bellum, and occipital lobe	Ischemic or hemorrhagic	Holmes tremor	36	LEAD-DBS	Functional	Brain Genomics Superstruct Project (GSP) with 1000 healthy participants	7
Kim	2019	Cross-sectional	Case reports	89	NR	NR	NR	NR	NR	NR	NR	Hallucinations	89	SPM	Functional	Brain Genomics Superstruct Project (GSP) with 1000 healthy participants	6
Darby	2018	Cross-sectional	Case reports	40	9–62	NR	NR	Heterogenous	Heterogenous	NR	Heterogenous	Criminal behaviour	17	FSL and LEAD-DBS	Functional	Brain Genomics Superstruct Project (GSP) with 1000 healthy participants	7
Joutsa	2018	Cross-sectional	Case reports	11	59–90	4	Until 2016	Stroke	Stroke	Heterogenous	Ischemic	Essential tremor	11	FSL	Functional	Brain Genomics Superstruct Project (GSP) with 1000 healthy participants	8
Joutsa	2018	Cross-sectional	Case reports	29	16–83	13	Until 2017	Heterogenous	Stroke, Haemorrhage, tumor, Hypoxia	Heterogenous	Ischemic, Haemorrhagic, tumoral	Parkinsonism	29	FSL and LEAD-DBS	Functional	Brain Genomics Superstruct Project (GSP) with 1000 healthy participants	8
Darby	2017	Cross-sectional	Case reports	17	9–62	NR	NR	Heterogenous	Heterogenous	NR	Heterogenous	Delusional misidentifications	17	FSL	Functional	Brain Genomics Superstruct Project (GSP) with 1000 healthy participants	7
Fasano	2016	Cross-sectional	Case reports	14	35–80	3	1993–2013	Heterogenous	Stroke, Haemorrhage, tumor	Heterogenous	Ischemic, Haemorrhagic, tumoral	Freezing of gait	14	FSL	Functional	Brain Genomics Superstruct Project (GSP) with 98 healthy participants	8
Laganiere	2016	Cross-sectional	Case reports	29	60.2	NR	Until 2014	Stroke	Stroke	Cortex (*n*=8), STN (*n*=8), putamen (*n*=6), caudate (*n*=5), midbrain (*n*=1), and subcortical white matter (*n*=1)	Ischemic	Hemichorea-hemiballismus	29	FSL	Functional	Brain Genomics Superstruct Project (GSP) with 98 healthy participants	8
Boes	2015	Cross-sectional	Private and case reports	23	61 ± 19	NR	NR	Heterogenous	Heterogenous	Heterogenous	Heterogenous	Peduncular hallucinosis	23	FSL	Functional	Brain Genomics Superstruct Project (GSP) with 98 healthy participants	8
Darby	2018	Cross-sectional	Case reports	28	67.3	NR	NR	Stroke and Hemorrhage	Stroke and Hemorrhage	Anterior cingulate cortex (ACC) (21% of cases), globus pallidus (29%), thalamus (25%), caudate (18%), and brainstem (11%)	Ischemic, Haemorrhagic	Akinetic mutism	28	FSL and LEAD-DBS	Functional	Brain Genomics Superstruct Project (GSP) with 1000 healthy participants	8
Ferguson	2019	Cross-sectional	Case reports	53	57.5 ± 13	34	NR	Heterogenous	Heterogenous	Heterogenous	Heterogenous	Amnesia	53	FSL	Functional	Brain Genomics Superstruct Project (GSP) with 1000 healthy participants	8
Ferguson	2021	Cross-sectional	Private	193	NR	NR	NR	Brain tumor resection and brain injury	Tumor and head truama	Heterogenous	Heterogenous	Spirituality and religiosity	193	FSL and 3D slicer	Functional	Brain Genomics Superstruct Project (GSP) with 1000 healthy participants	7
Fischer	2016	Case-control	Private	36	57.3 6 16.5	NR	NR	Heterogenous	Heterogenous	Pons, midbrain, and Medulla	Heterogenous	Coma	12	FSL	Functional	Brain Genomics Superstruct Project (GSP) with 98 healthy participants	8
Herbert	2019	Cross-sectional	Private	14	40.07 ± 11.08	6	2011–2018	Brain tumor resection	Glioma	Pons, midbrain, and Medulla	Tumoral and resection	Bodily awareness	14	MATLAB and SPM	Functional	Local data of 18 healthy participants	8
Jimenez-Marin	2022	Cross-sectional	Private	54	68.7	29	NR	Stroke	Stroke	Heterogenous	Ischemic, Haemorrhagic	Poststroke sensorimotor outcomes	54	MATLAB and SPM	Functional and structural	Brain Genomics Superstruct Project (GSP) with 1000 healthy participants	8
Padmanabhan	2019	Case-control	Private	358	59.3	86	NR	Ischemic stroke, intracerebral hemorrhage, and penetrating traumatic brain injury	Ischemic stroke, intracerebral hemorrhage, and penetrating traumatic brain injury	Heterogenous	Heterogenous	Depression	58	FSL	Functional	Brain Genomics Superstruct Project (GSP) with 1000 healthy participants	7
Kletenik	2022	Case-control	Case reports	69	NR	NR	NR	Tumor, Stroke and Hemorrhage	Heterogenous	Heterogenous	Heterogenous	Blindsight	34	FSL and 3D slicer	Functional	Brain Genomics Superstruct Project (GSP) with 1000 healthy participants	8
Siddiqi	2021	Cross-sectional	Private	713	NR	NR	NR	Stroke, Parkinson's disease, Epilepsy, Penetrating traumatic brain injury, and Major depressive disorder	Stroke, DBS, TMS	Heterogenous	Heterogenous	Neuropsychiatric disease	713	FSL and MATLAB	Functional	Brain Genomics Superstruct Project (GSP) with 1000 healthy participants	8
Alves	2022	Cross-sectional	Case reports	67	NR	NR	NR	Stroke	Stroke	Heterogenous	Heterogenous	Delusions of space	67	FSL	Structural	Human Connectome Project with 178 healthy participants	7
Conrad	2022	Cross-sectional	Case reports	10	NR	NR	NR	Stroke	Stroke	Anterior long insular gyrus (IV) and posterior long insular gyrus (V), and extended to the anterior insula.	Ischemic	Cortical vertigo	10	FSL	Functional and structural	Human Connectome Project with 178 healthy participants for structural and 100 healthy participants for functional	8
Cotovio	2022	Cross-sectional	Case reports	687	NR	NR	NR	Heterogenous	NR	Wide range of cortical and subcortical areas	Heterogenous	Mania	56	FSL	Functional	Human Connectome Project with 937 healthy participants and Max Planck Institute (MPI)-Leipzig Mind Brain Body with 189 healthy participants	6
Dulyan	2022	Longitudinal	Private	62	53.7	28	NR	Stroke	Stroke	Thalamus, putamen, caudate, pallidum, hippocampus, amygdala, nucleus accumbens, insula, subcallosal cingulate, paracingulate, and parahippocampal areas	Ischemic and Hemorrhagic	Motor dysfunction	62	MATLAB	Structural	Human Connectome Project with 163 healthy participants	7
Jiang	2023	Cross-sectional	Private	167	58.1	0	2003–2006	Brain injury	Brain injury	Heterogenous	NR	Emotion Regulation	167	FSL	Functional	Brain Genomics Superstruct Project (GSP) with 1000 healthy participants	7
Kolskar	2022	Cross-sectional	Private	102	66.3	26	NR	Stroke	Stroke	Heterogenous	Ischemic and Hemorrhagic	Cognitive impairment	102	MATLAB	Structural	Human Connectome Project with 170 healthy participants	8
Li	2023	Cross-sectional	Case reports	23	NR	13	NR	Stroke	Stroke	Heterogenous	Ischemic and Hemorrhagic	Vertigo	23	FSL and LEAD-DBS	Functional	Brain Genomics Superstruct Project (GSP) with 1000 healthy participants	8
Ulrichsen	2021	Cross-sectional	Private	239	65.8	68	NR	Stroke	Stroke	Heterogenous	Ischemic and Hemorrhagic	Fatigue	84	FSL	Structural	Human Connectome Project with 170 healthy participants	8
Rosenzopf	2022	Cross-sectional	Private	101	57.7	37	NR	Stroke	Stroke	Left hemisphere	Ischemic and Hemorrhagic	Limb apraxia	31	FSL	Structural	IIT Human Brain Atlas	7
Siddiqi	2023	Cross-sectional	Private	281	48.7	205	2015–2017	Multiple sclerosis	Multiple sclerosis	Heterogenous	MS lesions	Depression	281	FSL and MATLAB	Functional	Brain Genomics Superstruct Project (GSP) with 1000 healthy participants	8
Sotelo	2019	Cross-sectional	Private	13	63.4	7	NR	Stroke	Stroke	Heterogenous	Ischemic and Hemorrhagic	Motor impairment	13	FSL and MATLAB	Structural	Private	7
Weaver	2023	Cross-sectional	Private	553	69	233	NR	Stroke	Stroke	Heterogenous	Ischemic and Hemorrhagic	poststroke depressive symptoms	553	BCBtoolkit	Structural	Private	7

NR, Not Reported; NOS, Newcastle-Ottawa Scale; SPM, Statistical Parametric Mapping; FSL, FMRIB Software Library.

### Functional lesion network mapping

Non-motor symptoms were found to be associated with fronto-parieto-temporal networks (24), sensorimotor and ventral attention networks (29), and the thalamic mediodorsal nucleus (14). Executive function deficits demonstrated connectivity with the anterior cingulate cortex, dorsomedial prefrontal cortex, and frontoparietal network (16). Symptoms such as prosopagnosia, anosognosia for hemiplegia, and diminished mind-wandering revealed connections to the left frontal cortex, anterior cingulate cortex, right fusiform face area (10), right posterior hippocampus (33), and left inferior parietal lobule (35) respectively.

Symptoms such as loss of consciousness, mania, and delusional misidentifications were associated with connectivity to the dorsal brainstem (37), right orbitofrontal cortex, right inferior temporal gyrus, right frontal pole (27), and left retro splenial and right frontal cortex (7). Additionally, hallucinations were linked to the extrastriate visual cortex (4) and to the cerebellar vermis, inferior cerebellum, and right superior temporal sulcus (5). Lesions causing autoscopic phenomena showed functional connections to the bilateral temporoparietal junction (23).

Cortical vertigo showed associations with connectivity to the posterior insula (52). Lesions causing obsessive–compulsive disorder (OCD) and depression were linked to the dorsal anterior cingulate cortex and left dorsolateral prefrontal cortex (11, 30).

Findings indicate that behavioral deficits were better predicted by direct measures of functional MRI connectivity than indirect functional disconnection (38). Moreover, Darby et al. (15) identified that criminal behavior was associated with a shared network encompassing the inferior orbitofrontal cortex, anterior temporal lobes, and intraparietal sulcus (Table 2).

**Table 2:** LNM findings of the included studies.

Studied symptom or disorder	Number of subjects with symptom	Lesion network mapping findings	Author	Year
Addiction	129	1- Lesions disrupting smoking addiction occurred in many different brain locations but were characterized by a specific pattern of brain connectivity. This pattern involved positive connectivity to the dorsal cingulate, lateral prefrontal cortex, and insula and negative connectivity to the medial prefrontal and temporal cortex, 2- This circuit was reproducible across independent lesion cohorts, associated with reduced alcohol addiction risk, and specific to addiction metrics. Hubs that best matched the connectivity profile for addiction remission were the paracingulate gyrus, left frontal operculum, and medial fronto-polar cortex	Joutsa	2022
Akinetic mutism	28	Brain network defined by functional connectivity to the anterior cingulate cortex	Darby	2018
Amnesia	53	Over 95% of amnesia-causing lesion locations were functionally connected to a single location in the hippocampus	Ferguson	2019
Anosognosia for hemiplegia	25	Right posterior hippocampus showed significantly greater normative lesion connectivity for anosognosia for hemiplegia	Klingbeil	2020
Asymmetric step length	39	Functional: At least 85% of lesions showed functional network overlap in the bilateral frontal lobe. Structural: The overlap of lesion-derived structural networks was high (85%) and occurred specifically within the corona radiata of the lesional hemisphere	Kyeong	2020
Autoscopic phenomena	26	1- Autoscopic phenomena localize to bilateral temporo-parietal junction, 2- Out-of-body-experience resulted from a brain network connected to bilateral angular gyrus, right precuneus, and right inferior frontal gyrus, differing from autoscopic hallucination with a brain network connected to bilateral precuneus, inferior temporal gyrus, and cerebellum, 3- Heautoscopy resulted from a brain network connected to left inferior frontal gyrus, left insula and left parahippocampus	Blondiaux	2021
Behavioral deficits	123	This principal component functional disconnection approach localized mainly cortical voxels of high signal-to-noise; and it yielded networks with high anatomical specificity, and strong behavioural correlation	Pini	2021
Behavioral deficits	132	Functional: Prediction from indirect functional disconnection was scarce or negligible except for the right visual field deficits. Prediction from direct measures of functional MRI functional connectivity in a subset of patients was clearly superior to indirect functional disconnection. Structural: The indirect estimation of structural connectivity damage successfully predicted behavioural deficits post-stroke to a level comparable to lesion information. However, indirect estimation of functional disconnection did not predict behavioural deficits	Salvalaggio	2020
Blindsight	34	The functional connectivity observed between the lesion locations and the ipsilesional medial pulvinar was found to be significantly associated with blindsight. However, no significant differences in connectivity were identified with respect to other brain regions, which have been previously implicated in blindsight	Kletenik	2022
Bodily awareness	14	The resection cavity maps in patients with body awareness disorders exhibited robust connectivity to a sensorimotor network consisting of the antero-dorsal precuneus, paracentral lobule, supplementary motor area, superior parietal lobule, supramarginal gyrus, insula, and premotor cortex	Herbert	2019
Cerebellar cognitive affective syndrome	48	1- Cerebellar region most associated with cerebellar cognitive affective syndrome was functionally connected to the thalamic mediodorsal nucleus, 2- higher connectivity between lesion location and the mediodorsal nucleus predicts cerebellar cognitive affective syndrome occurrence	Albazron	2019
Cognitive and motor outcomes	593	The Boston Naming Test linked with most results converging on a fronto-parieto-temporal network. Two principal components were linked to the Token Test, and these seeds also converged primarily on a fronto-parieto-temporal network. Results based on the delayed recall trial from the Rey Auditory Verbal Learning Test identified only two networks: a lateral occipital-precuneate network, and a network spanning primary and secondary visual cortices. Functional lesion network mapping performed best for the prediction of language deficits, and structural lesion network mapping performed best for the prediction of motor deficit	Bowren	2022
Cognitive impairment	102	An analysis of the disconnectome illustrated that increased disconnection in the right insular and frontal operculum, superior temporal gyrus, and putamen was related to a decline in MoCA performance, suggesting that lesions in regions linked to these brain regions are more likely to result in cognitive impairment	Kolskar	2022
Coma	12	A small region in the rostral dorsolateral pontine tegmentum is significantly associated with coma-causing lesions and is functionally connected to the ventral anterior insula and pregenual anterior cingulate cortex	Fischer	2016
Cortical vertigo	10	Structural disconnection: The fronto-insular tracts, specifically fronto-insular tracts 4 and 5, facilitate connections between the parietal operculum and the posterior regions of the insula as well as the inferior fronto-occipital fascicle (IFOF). Additionally, the third division of the superior longitudinal fascicle (SLF III) was affected to a greater extent. It is important to note that in cases with vertigo, two white matter tracts were disconnected, namely the fibers of the splenium of the corpus callosum in all 10 cases and posterior segments of the arcuate fascicle in 9 out of 10 cases. These white matter tracts were not affected in lesions without vertigo. Functional: The functional connectivity networks (FCNs) share common subcortical components, which include the vestibular nuclei (VN) and the cerebellar vestibular and ocular motor representations located in lobules IX (nodulus, uvula) and X (flocculus/paraflocculus). In addition, cortical network hubs comprise the PIVC, the posterior insular cortex (PIC) and the adjacent superior temporal gyrus, as well as vestibular multisensory areas located further away, such as the ventral intraparietal area (VIP), motion-sensitive areas MT+ in the temporal lobe, and cingulate visual sulcus (CSv), along with the ocular motor areas of the parietal (lateral parietal area—LIP) and frontal lobes (frontal eye fields, FEF, and dorsolateral prefrontal cortex, DLPFC)	Conrad	2022
Criminal behaviour	17	1- All 17 lesions temporally associated with criminal behavior were functionally connected (i.e., positively correlated) to the inferior orbitofrontal cortex and anterior temporal lobes, and most (16 of 17) were connected to the vmPFC and nucleus accumbens. 2- All 17 lesions were functionally connected (i.e., negatively correlated) with the intra parietal sulcus, and 15 of 17 were functionally connected with the dorsolateral prefrontal cortex	Darby	2018
Delusional misidentifications	17	1- All 17 lesion locations were functionally connected to the left retrosplenial cortex, 2- Similarly, 16 of 17 lesion locations were functionally connected to the right frontal cortex	Darby	2017
Delusions of space	67	Lesions caused delusion of space were assocaited with disconnection right ventrolateral prefrontal and right temporal cluster	Alves	2022
Depression	58	There was a notable increase in connectivity between the lesions of depressed individuals and a specific area of the left dorsal lateral prefrontal cortex when compared to the lesions of non-depressed individuals	Padmanabhan	2019
Depression	281	The present study demonstrated that the functional connectivity of multiple sclerosis (MS) lesion locations with our pre-determined depression circuit (involving the dorsolateral prefrontal cortex, subgenual cingulate, and ventromedial prefrontal cortex) was significantly linked with the severity of depression in MS patients. Furthermore, this association was observed specifically in relation to depression and not with other symptoms associated with MS	Siddiqi	2023
Emotion Regulation	167	The construction of the brain network for regulating emotions utilizing lesion-related information was characterized by the functional association with the left ventrolateral prefrontal cortex	Jiang	2023
Epilepsy	51	Greatest functional connectivity overlap was in Frontoparietal Network, Ventral Attention Network, and the Limbic Network—with percentage volume overlap of 19.5%, 19.1%, 19.1%, and 12.5%, respectively	Mansouri	2020
Essential tremor	11	All 11 lesion locations were connected to the bilateral thalamus, bilateral cerebellum, left globus pallidus, and left putamen	Joutsa	2018
Executive Function	15	Thalamic lesion sites associated with more severe deficits in executive function showed stronger functional connectivity with anterior cingulate cortex, dorsomedial prefrontal cortex, and frontoparietal network, compared to thalamic lesions not associated with executive dysfunction	Hwang	2020
Fall risk	160	There was significant correlations between the percentage of lesion related disruption of the dorsal attention network and Physiological Profile Assessment (PPA) score; and between disruption of both the sensorimotor and ventral attention networks with foam sway. There were no significant associations with floor sway or gait speed	Crockett	2022
Fatigue	84	There was no significant associations between the disconnectome maps and the clinical measures	Ulrichsen	2021
Foreign accent syndrome	25	At least 80% of lesions showed network overlap in the bilateral lower and middle por tions of the precentral gyrus and in the medial frontal cortex	Higashiyama	2021
Freezing of gait	14	(13/14) of lesions were functionally connected to a focal area in the dorsal medial cerebellum	Fasano	2016
Global cognition	160	The visual, ventral attention, and frontoparietal networks had significant overlap with the lesion network. After controlling for multiple comparisons, level of lesion network overlap with both the sensorimotor network and ventral attention network was significantly correlated with MoCA score. Thus, the disruption to the sensorimotor and ventral attention networks, associated with the poorer global cognition	Crockett	2021
Hallucinations	89	Hallucinations was defined by connectivity to the cerebellar vermis, inferior cerebellum, and the right superior temporal sulcus	Kim	2019
Hemichorea-hemiballismus	29	At least 90% of lesions showed network overlap in the posterolateral putamen	Laganiere	2016
Holmes tremor	36	All lesion locations were connected to a common brain circuit with nodes in the red nucleus, thalamus, globus pallidus, and cerebellum	Joutsa	2019
Idiopathic cervical dystonia	25	Positive connectivity to the cerebellum and negative connectivity to the somatosensory cortex were specific markers for cervical dystonia	Corp	2019
Infantile Spasms	74	Infantile spasms connected to the globi pallidi and cerebellar vermis	Cohen	2021
Limb apraxia	31	The present study identified significant pathological changes in the white matter of a densely interconnected fronto-temporo-parietal network consisting of both short and long distance association fibers. Accordingly, the results imply that the divergent topographical outcomes reported in prior lesion mapping investigations may not solely stem from variations in research methodology but also from the limitations inherent in univariate topographical mapping techniques to reveal the complex structural praxis network	Rosenzopf	2022
Loss of consciousness	171	The map of regions anticorrelated to the dorsal brainstem thus defines a distributed brain circuit that, when damaged, is most likely to cause loss of consciousness. This circuit showed a slight posterior predominance and had peaks in the bilateral claustrum	Snider	2020
Mania	15	Lesion locations showed a unique pattern of functional connectivity to the right orbitofrontal cortex, right inferior temporal gyrus, and right frontal pole	Cotovio	2020
Mania	56	The researchers evaluated the effect of utilizing distinct connectomes on the outcomes of lesion network mapping for mania. Their findings indicated that the conclusions were dependable and uniform, regardless of the specific connectome employed for the analysis	Cotovio	2022
Mind-wandering	29	Lesion network mapping analyses showed the strongest association of reduced mind-wandering with the left inferior parietal lobule	Philippi	2020
Motor dysfunction	62	The isolated lesions reflect a symmetrical but predominantly right-sided lack of connection, with a greater degree of overlap noted in the ventral visual pathways, internal capsule, and perisylvian white matter	Dulyan	2022
Motor impairment	13	They found significantly reduced indirect connectivity in the frontal and parietal lobes, ipsilesional subcortical regions and bilateral cerebellum after stroke	Sotelo	2019
Neuropsychiatric disease	713	The severity of depression was found to be associated with specific lesion and stimulation sites, which were connected to a consistent brain circuit across multiple datasets. The circuits derived from lesions, deep brain stimulation, and transcranial magnetic stimulation were comparable, and the circuits derived from patients with major depression and those with other diagnoses were similar as well. The connectivity of these circuits was predictive of the out-of-sample antidepressant efficacy of deep brain stimulation and transcranial magnetic stimulation sites. Furthermore, a separate analysis involving 29 lesions and 95 stimulation sites identified a unique circuit for the motor symptoms of Parkinson's disease	Siddiqi	2021
Obsessive compulsive disorder	11	Lesion functional connectivity with two distinct frontal regions, the dorsal anterior cingulate cortex and the left dorsolateral pre frontal cortex was highly correlated with individual symptom improvement	Germann	2021
Parkinsonism	29	Lesion locations causing parkinsonism were functionally connected to a common network of regions including the midbrain, basal ganglia, cingulate cortex, and cerebellum	Joutsa	2018
Peduncular hallucinosis	23	22 of 23 lesions were negatively correlated with extrastriate visual cortex	Boes	2015
poststroke depressive symptoms	553	Utilizing disconnectome-based analyses, the results of this study demonstrated that disruptions in the white matter of the right parahippocampal region, as well as the right thalamus and pallidum, and the right anterior thalamic radiation were significantly linked to the manifestation of depressive symptoms following a stroke	Weaver	2023
Poststroke sensorimotor outcomes	54	Functional: The functional lesion-disconnectivity technique produced the highest behavioral association local network maps, which indicated that the brainstem (specifically the pons), left supramarginal gyrus (in the portion overlapping with the secondary somatosensory cortex), left thalamus, bilateral superior frontal cortex (in the portion overlapping with the premotor cortex and supplementary motor area), left inferior parietal cortex, and right precentral cortex (in the portion overlapping with the primary motor cortex and primary sensory cortex) were involved in both unimodal and multimodal associations. Structural: The top behavioral association maps generated by lesion network mapping techniques using structural lesion-disconnectivity analysis showed that several major tracts, including the forceps major, left frontal aslant tract, left anterior thalamic radiation, bilateral superior longitudinal fasciculus, and bilateral optic radiation, were heavily involved in both unimodal and multimodal analyses	Jimenez-Marin	2022
Prosopagnosia	44	All 44 lesion locations were functionally connected, through negative correlation, with regions in the left frontal cortex, and anterior cingulate cortex, and also, positevly correlated with right fusiform face area	Cohen	2019
Semantic Aphasia	23	There was significant overlap in the distinct patterns of structural and functional disconnection	Souter	2022
Spirituality and religiosity	193	The peak association with changes in spiritual acceptance was connectivity between lesion locations and the periaqueductal grey	Ferguson	2021
Tics	22	Tic-inducing lesions mapped to a common network map, which comprised the insular cortices, cingulate gyrus, striatum, globus pallidus internus, thalami, and the cerebellum	Ganos	2022
Vertigo	23	Analysis demonstrated that the functional connectivity established between the locations of the lesions and the bilateral ventral posterior insula was highly sensitive (observed in 22 out of a total of 23 lesions) and precise in diagnosing vertigo resulting specifically from lesions	Li	2023

LNM, Lesion Network Mapping.

Movement disorders, such as essential tremor, Parkinsonism, freezing of gait, and Holmes tremor, were linked to networks involving the cerebellum and thalamus (32), midbrain, basal ganglia, cingulate cortex, and cerebellum (19), and red nucleus, thalamus, globus pallidus, and cerebellum (17), respectively.

Hemichorea hemiballism, cervical dystonia, and increased fall risk were associated with the posterolateral putamen (20), cerebellum and somatosensory cortex (26), and the dorsal attention network (28), respectively. Additionally, asymmetric step length after a unilateral stroke demonstrated functional connectivity to the dorsolateral prefrontal cortex (18).

Speech disorders such as semantic aphasia, foreign accent syndrome, and apraxia of speech were associated with distinctive patterns of structural and functional disconnection (58), and networks involving the bilateral lower and middle portions of the precentral gyrus and medial frontal cortex (31).

**Figure 3:**
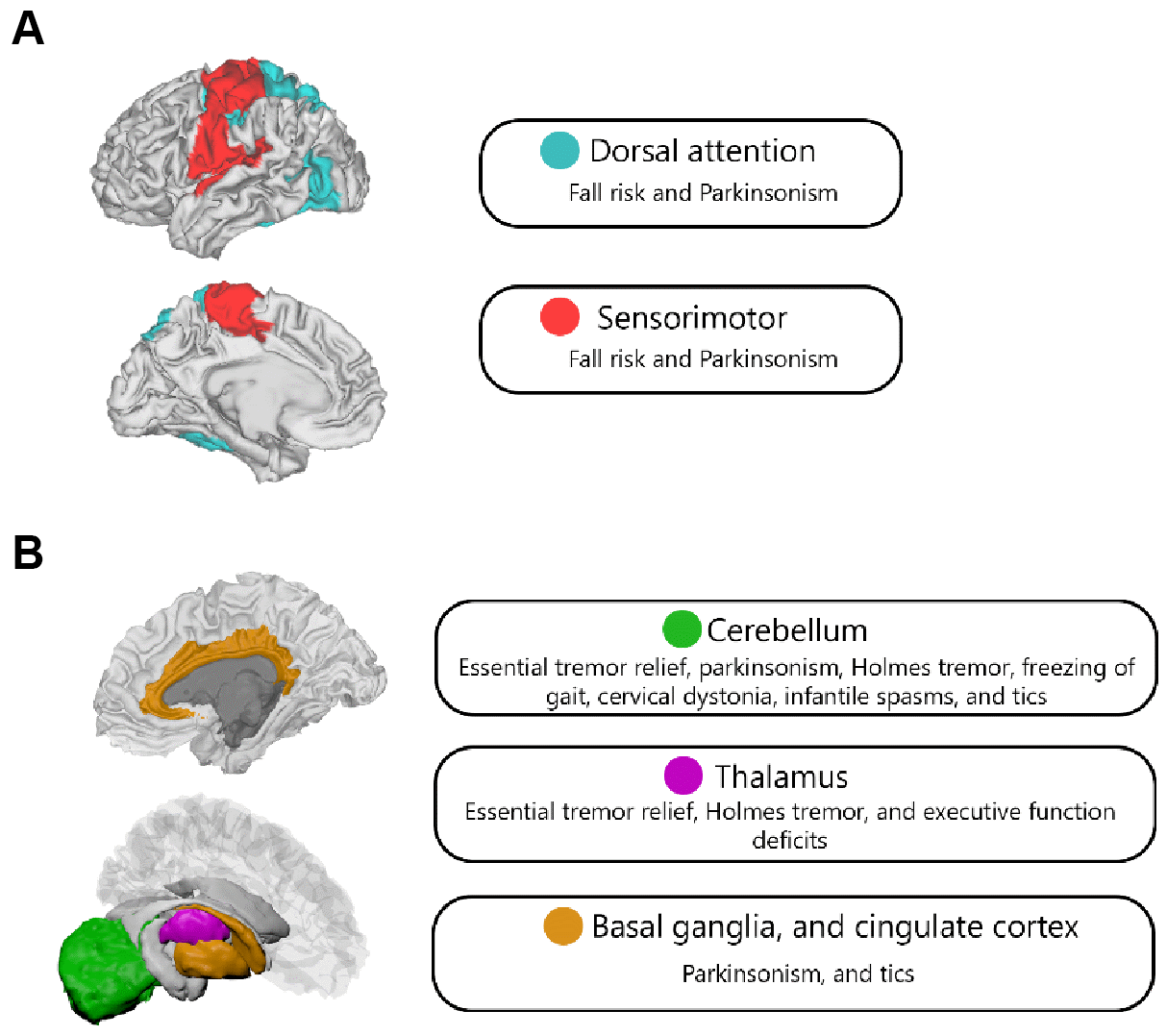
Network **(A)** and region **(B)** overlap across different psychiatric and neurological disorders in functional LNM studies.

Studies using functional connectivity revealed several region and network overlaps across different movement or psychiatric disorders (Figure 3 and Table 3). The cerebellum appeared in a common network for conditions such as essential tremor relief (32), parkinsonism (19), Holmes tremor (17), freezing of gait (8), cervical dystonia (26), and tics (6). It also showed functional connections to tuberous sclerosis lesions in children with infantile spasms (59). The thalamus demonstrated involvement in a common network for essential tremor relief (32) and Holmes tremor (17), and showed significant functional connectivity with lesions associated with executive function deficits (16). The midbrain, basal ganglia, and cingulate cortex were connected to lesions causing parkinsonism (19) and were part of the common network implicated in tics (6).

**Table 3:** Common brain regions and networks across neurological and psychiatric disorders.

Common brain regions	
Anterior Cingulate Cortex	Involved in executive function deficits, prosopagnosia, and anosognosia for hemiplegia
Left Frontal Cortex	Connected to prosopagnosia, anosognosia for hemiplegia, and reduced mind-wandering
Cerebellum	Common in networks involving essential tremor, Parkinsonism, Holmes tremor, freezing of gait, cervical dystonia, tics, hallucinations, and infantile spasms in children with tuberous sclerosis
Thalamus	Involved in essential tremor, Holmes tremor, and executive function deficits

Common networks	
Frontoparieto-temporal Network	Linked to non-motor symptoms
Sensorimotor Network	Connected to non-motor symptoms and cognitive impairment
Frontoparietal Network	Associated with executive function deficits and epileptogenic mass lesions
Dorsal Attention Network	Linked to Parkinsonism and fall risk

The dorsal attention network was significantly correlated with the Physiological Profile Assessment (PPA) score, which measures fall risk in elderly individuals (28), and demonstrated functional connectivity related to focal brain lesions causing parkinsonism (19). It was also part of the frontoparietal network, which showed functional connectivity overlap in regions connected to epileptogenic mass lesions (34). Moreover, the sensorimotor network showed significant overlap in lesions causing cognitive impairment assessed by the MoCA score (29), and was connected to resection cavity maps in patients with body awareness disorders (42).

### Structural lesion network mapping

The emerging evidence underscores the reciprocal nature of structural and functional Lesion Network Mapping (LNM) in elucidating the nexus between cerebral lesions and cognitive functionality (24, 29, 51). Structural mapping has pinpointed specific regions, such as the right insular and frontal operculum, superior temporal gyrus, and putamen, whose impairment tends to precipitate cognitive deficits (51). Concurrently, functional mapping has demystified the distinctive brain networks correlated with various cognitive faculties, exemplified by the fronto-parieto-temporal network (24).

The intricate neural networks engaged in spatial perception have been brought to light, with aberrations in the right ventrolateral prefrontal and right temporal clusters linked to spatial delusions (47). Importantly, the integral role of the left retro splenial and right frontal cortex in spatial information processing has been underscored, underlining their pivotal contribution to spatial cognition and awareness (48).

Furthermore, the studies spotlight the necessity of incorporating structural connectivity into the neurological evaluation of stroke sequelae for optimal therapeutic results (38). Structural LNM exhibited superior reliability in forecasting post-stroke behavioral repercussions compared to its functional counterpart. However, there were instances where direct measures of functional connectivity outperformed, underscoring the indispensable role of a holistic evaluation of both structural and functional connectivity to tailor personalized therapeutic interventions for stroke survivors.

The critical role of the frontal lobe in gait regulation was reaffirmed in the context of motor and speech disorders (18). The choice of LNM methodology appears to be contingent upon the specific functional impairment in question, with functional mapping outperforming in the domain of language deficits, while structural mapping took the lead for motor deficits (24). Both techniques unveiled significant pathways, indicating that diverse lesion types might disrupt distinct neural circuits, thus informing rehabilitative strategies post-stroke (45). For example, the left anterior thalamic radiation and bilateral superior longitudinal fasciculus were highlighted as significant pathways by both methodologies (45).

Lastly, the studies emphasized the significance of comprehending the intricate structural praxis network in limb apraxia patients (53). The indirect structural disconnection method discerned significant pathological alterations in the white matter within an extensively interconnected fronto-temporo-parietal network, incorporating both short and long-distance association fibers (53). This revelation suggests that the disparate topographical outcomes reported in earlier lesion mapping studies might not exclusively arise from methodological variations but could also be attributed to the inherent limitations of univariate topographical mapping techniques.

## Discussion

This review consolidates and critically assesses existing knowledge on the application of Lesion Network Mapping (LNM) methods in the diagnosis and treatment of neurological and psychiatric disorders. Our findings underscore the complementary role of functional and structural LNM, emphasizing their potential utility in improving therapeutic outcomes in neurological afflictions. Our analysis draws attention to certain brain regions and networks integral to specific neurological domains. For instance, the fronto-parieto-temporal network emerged as pivotal to cognitive functioning, while the dorsal anterior cingulate cortex and the left dorsolateral prefrontal cortex demonstrated considerable relevance for obsessive–compulsive disorder. These insights indicate that LNM may aid in pinpointing viable targets for neuromodulation interventions across various neurological disorders. The examined studies also underlined the indispensable role of LNM in deciphering the intricacies of motor and speech disorders. These studies highlighted the use of LNM in identifying brain regions and neural pathways implicated in diverse movement disorders, such as Parkinsonism, Essential Tremor, and Holmes Tremor. Consequently, we can anticipate LNM’s instrumental role in informing neuromodulation strategies tailored to specific motor and speech disorders, such as cervical dystonia and hemichorea hemiballismus. Our results suggest that the choice of LNM method should align with the specific type of functional deficit under consideration. Since the advent of LNM studies in 2015, numerous investigations have successfully employed this technique to elucidate how alterations in functional and structural networks can account for symptoms post focal brain lesions.

Moreover, our analysis identified several overlaps between different regions and networks implicated in diverse psychiatric and neurological disorders. Both structural (51) and functional LNM (24, 29) pinpointed the fronto-parieto-temporal network as integral to cognitive function, thereby asserting a potent correlation between this network and cognitive impairment. Furthermore, studies revealed that damage to the right ventrolateral prefrontal and right temporal cluster (47), coupled with alterations in the left retro splenial and right frontal cortex (7), could precipitate spatial perception deficits and engender delusions of space. Intriguingly, both functional LNM (54) and structural disconnectome-based analyses (57) associated the left dorsolateral prefrontal cortex with depression. This concurrence insinuates a common neural foundation for depressive symptoms, stressing the necessity of considering both structural and functional connectivity when attempting to comprehend the neurological underpinnings of varying disorders and enhancing treatment outcomes. The complex neural networks implicated in these disorders can guide the formulation of tailored treatment strategies and facilitate a deeper understanding of their neurobiological origins. By delineating these overlaps and parallels, we underscore the interwoven nature of the brain and its instrumental role across an array of disorders.

Our study unearthed a significant overlap within the thalamus for essential tremor relief, Holmes tremor, and executive function deficits. This intriguing finding challenges the conventional practice of symptom localization that ascribes particular symptoms to specific, isolated brain regions. Instead, it accentuates the importance of investigating the complex interactions among various brain regions and acknowledges the distributed nature of neural processing (4, 24). This shared neural substrate within the thalamus proposes potential unified therapeutic targets for these conditions, underscoring the imperative for a more holistic approach when studying neurological and psychiatric disorders. By harmonizing the strengths of both conventional localization and network-based perspectives, researchers can attain a comprehensive understanding of the brain’s structure and function, ultimately leading to more effective treatment strategies for a multitude of disorders.

The neurobiological mechanisms underlying brain disorders and the localization of brain function are traditionally explored by identifying abnormalities within the brain and contrasting them with matched controls, as exemplified by the study of hemiparesis following stroke lesions. However, this process becomes complex when the lesion is located in an unanticipated region or when numerous heterogeneous lesions are observed in disparate locations. For instance, hemiparkinsonism patients with varied causal lesions outside the nigrostriatal tract were found to map onto a common network (19). Moreover, most neurological and psychiatric symptoms cannot be ascribed to a single region; instead, they involve a network of interconnected areas. As such, LNM has proven to be a significant advance over traditional lesion analysis, enabling the localization of symptoms across different lesion locations, a task that was previously unachievable (60).

Focal brain lesions have been observed to modify resting functional connectivity and reduce the variability of neural states, thereby limiting the brain’s ability to process information (59, 61, 62). Empirical evidence from previous studies has indicated that alterations in functional connectivity following focal brain lesions are not restricted to a singular network but engage numerous regions (63). Strokes tend to affect white matter and subcortical regions more often than the cortex. Given that white matter contains numerous fiber pathways, strokes within these regions can result in widespread alterations (59, 64).

The pioneering study employing LNM was conducted by Boes et al. (4), investigating peduncular hallucinosis subsequent to subcortical lesions. These lesions were hypothesized to disrupt the extrastriate visual cortex, despite their heterogeneous locations. The researchers found that 22 out of 23 lesions demonstrated a negative correlation with the extrastriate visual cortex. Moreover, a study by Kim et al. discovered that lesions causing hallucinations localized to a shared brain network, encompassing the cerebellar vermis, inferior cerebellum, and the right superior temporal sulcus (5). Following the promising results of this inaugural LNM study, the method was applied to elucidate long-standing neurological enigmas characterized by heterogeneous lesions dispersed across different regions (6, 18, 31).

Considering the comparable effects of deep brain stimulation (DBS) and therapeutic lesions, a promising avenue for LNM involves leveraging the identified regions associated with disorders or common networks linked to beneficial brain lesions as therapeutic targets for DBS (65, 66). A pertinent example is the observed clinical improvement in patients where DBS electrode connectivity was in the claustrum, which was also identified as a shared network for lesions causing parkinsonism *via* LNM (19). Further, the exploration of beneficial brain lesions that alleviate symptoms to pinpoint optimal DBS targets should extend to various neurological or psychiatric disorders. However, it’s important to note that beneficial brain lesions are exceedingly rare. A study by Joutsa et al. demonstrated that varied lesion locations resulting in essential tremor relief overlapped in a common network within the cerebellum and thalamus, known targets for deep brain stimulation in the management of tremors (32).

Furthermore, a recent study by the same group delved into brain lesions associated with addiction improvement, identifying the paracingulate gyrus and the left frontal operculum. The medial frontopolar cortex emerged as the best-matching connectivity profile for addiction remission (39). These discoveries could pave the way to optimal treatment targets for addiction disorders, lending support to ongoing neuromodulation trials (67). However, it is clear that additional research is required to understand how to extrapolate and interpret LNM findings to identify the most suitable therapeutic targets.

When lesion connections coincide within a single brain network, it’s reasonable to infer that the network has a causative role in symptom production. Nevertheless, the regions at the network’s core may not be crucial in symptom development (68). The correlation between symptoms and the central region of the network is gleaned from brain connectivity patterns. Consequently, regional associations gleaned from LNM must be appraised in comparison with functional neuroimaging results.

A prominent question within LNM pertains to the neurobiological mechanism at play when a network is disrupted by lesions. Although lesions induce dysfunction at their locations, the remote impact of lesions on functional loss in interconnected regions—dependent on the type of connection—remains a topic of debate (69). Dysfunctions in excitatory connections could lead to decreased activity, while disruption in inhibitory connections could result in increased activity (26). Furthermore, it remains uncertain whether the labeling of functional connectivity as positive or negative can indicate decreases or increases in activity, respectively.

LNM’s focus has largely been on the spatial aspect of symptoms induced by lesions, often neglecting the temporal component. Investigating the temporal aspect of symptoms is just as important, given that symptoms evolve over time due to recovery processes and dynamic changes post-injury (3).

## Limitation and future direction

While the development of LNM has yielded intriguing results, complete elucidation of neurological enigmas via this method alone remains a considerable distance away. More robust studies are necessary, particularly those utilizing prospective data that assess pre-and post-lesion symptoms. Salvalaggio et al. (38) have previously suggested that direct and indirect measures of functional networks may not be as sensitive to behavioral deficits compared to using structural disconnections, possibly due to the coincidence of structural disconnections with structural damage following lesions. However, Cohen et al. contended that the poor outcome of LNM in predicting behavioral deficits in Salvalaggio et al.’s study could be attributed to methodological considerations (25). Ferguson et al. (40) presented contrasting results, affirming the value of LNM in localizing behavioral deficits. These discrepancies underscore the need for future studies to compare and enhance LNM methods. Furthermore, the efficacy of LNM in identifying therapeutic targets should be examined by strategically placing DBS electrodes in the proposed hub of a network (69).

Cotovio et al. (27) probed the effects of utilizing distinct connectomes on LNM outcomes for mania, concluding that the findings remained consistent and reliable, regardless of the specific connectome used for analysis. Future directions could involve refining normative connectome atlases using higher resolution imaging, integrating results from various connectomes obtained during different tasks, and employing age-and sex-matched datasets for each patient. As functional and structural normative data often involve young, healthy individuals, employing normative lifespan data could yield deeper insights into brain function and enable more accurate symptom mapping. While most studies use normative connectomes, the exploration of disease-specific connectomes may provide more precise results.

Moreover, Bonkhoff et al. (70) discovered that women tend to experience more severe strokes than men, and a recent large-scale study highlighted the effect of sex on neuroimaging metrics over time (71). Thus, incorporating sex-specific normative data in LNM could enhance the precision of this approach.

While we have primarily considered functional and structural networks as relatively stable entities in this review, research increasingly shows that these networks are dynamically changing over time. These temporal fluctuations in connectivity patterns are believed to be crucial for flexible cognitive function, and disturbances in these dynamics have been linked to various neurological and psychiatric conditions. Incorporating these dynamic changes into LNM analyses could therefore potentially increase the sensitivity of the method to detect functional abnormalities linked to behavioral deficits. This integration of dynamics would be a novel addition to the methodology and potentially offers a new avenue for understanding brain function and dysfunction.

Another important discussion point to add is the development and application of machine learning techniques in the context of LNM. Recent advancements in machine learning and artificial intelligence (AI) present exciting opportunities for further refining and enhancing the predictive power of LNM. Machine learning models could be trained to predict the likelihood of specific deficits or symptom severity based on the observed lesion distribution and connectivity disruptions. Such models could provide clinicians with additional tools for prognosis and treatment planning, complementing the traditional, more qualitative approaches.

Lastly, the inter-individual variability in brain connectivity and anatomy should not be overlooked. Even among healthy individuals, there can be substantial differences in the structure and functional connectivity of the brain. This variability might influence the impact of a lesion on cognitive and behavioral function and might partly explain the heterogeneity observed in clinical outcomes following brain damage. Future studies could consider incorporating measures of inter-individual variability into the LNM framework to provide more personalized predictions about treatment outcomes. This could be especially relevant when considering DBS targets, as the optimal target might differ slightly between individuals due to this variability.

These proposed additions aim to enrich the current findings and provide a more comprehensive view of the applications and potential advancements in lesion network mapping.

## Methodological consideration

There are several important methodological considerations that should be addressed in LNM.

### Normalization and standardization

While LNM has proven to be a powerful tool in understanding brain disorders, it’s important to note that the results are highly dependent on the normalization and standardization of brain images. Subtle variations in these processes can lead to significant differences in results. Moreover, the choice of template used for spatial normalization can significantly impact the localization of brain regions and networks. Future studies should aim to implement standardized preprocessing pipelines to ensure the reliability and reproducibility of results.

### Limitations of normative connectomes

Most studies used normative connectomes in their LNM analyses. Although this approach provides a general framework for analyzing brain networks, it overlooks inter-individual variability. Individualized connectomes that account for each patient’s unique brain architecture may provide more precise mapping results and should be the focus of future research.

### Thresholding and statistical analysis

The manner in which network connections are thresholded and analyzed can greatly influence the results of LNM. The application of overly stringent thresholds may fail to capture weaker, yet potentially significant, network connections, whereas lenient thresholds may lead to false positive findings. The choice of statistical tests and corrections for multiple comparisons also plays a crucial role in the interpretation of results.

### Choice of parcellation scheme

The way in which the brain is divided into distinct regions, or parcels, can significantly impact the results of network analyses. Different parcellation schemes can yield differing, and sometimes conflicting, results. Hence, the choice of parcellation scheme should be carefully considered and justified in future LNM studies.

### Multimodal integration

The integration of multiple imaging modalities (e.g., structural MRI, functional MRI, diffusion tensor imaging) can provide complementary insights into the brain’s structure–function relationships. Despite this, many studies still focus on a single imaging modality, potentially missing important aspects of network functionality and connectivity.

While LNM presents an exciting tool in neurology and neuroimaging, attention to these methodological considerations is critical for the advancement of the field and to ensure the reliability and validity of findings.

## Conclusion

LNM offers solid findings in localizing a wide range of neuropsychiatric, behavioral, and movement disorders. Furthermore, LNM is anticipated to identify new treatment targets through symptom mapping. However, the veracity of these methodologies must be validated through more comprehensive prospective studies.

## Data availability statement

The original contributions presented in the study are included in the article/supplementary material, further inquiries can be directed to the corresponding author.

## Author contributions

FN and MA contributed to searching and writing and revising the manuscript. All authors contributed to the article and approved the submitted version.

## Funding

MA is funded by the EU-project euSNN European School of Network Neuroscience (MSCA-ITN-ETN H2020-860563).

## Conflict of interest

The authors declare that the research was conducted in the absence of any commercial or financial relationships that could be construed as a potential conflict of interest.

## Publisher’s note

All claims expressed in this article are solely those of the authors and do not necessarily represent those of their affiliated organizations, or those of the publisher, the editors and the reviewers. Any product that may be evaluated in this article, or claim that may be made by its manufacturer, is not guaranteed or endorsed by the publisher.
